# Methylome analysis in girls with idiopathic central precocious puberty

**DOI:** 10.1186/s13148-024-01683-1

**Published:** 2024-06-22

**Authors:** Stefania Palumbo, Domenico Palumbo, Grazia Cirillo, Giorgio Giurato, Francesca Aiello, Emanuele Miraglia del Giudice, Anna Grandone

**Affiliations:** 1 Department of Women’s and Children’s Health and General and Specialized Surgery, University of Campania “Luigi Vanvitelli”, Via Luigi De Crecchio 2, 80138 Naples, Italy; 2 Laboratory of Molecular Medicine and Genomics, Department of Medicine, Surgery and Dentistry SMS, University of Salerno, Salerno, Italy

**Keywords:** Epigenetics, Puberty, Differential methylation, Central precocious puberty, CPP, CpG

## Abstract

**Background:**

Genetic and environmental factors are implicated in many developmental processes. Recent evidence, however, has suggested that epigenetic changes may also influence the onset of puberty or the susceptibility to a wide range of diseases later in life. The present study aims to investigate changes in genomic DNA methylation profiles associated with pubertal onset analyzing human peripheral blood leukocytes from three different groups of subjects: 19 girls with central precocious puberty (CPP), 14 healthy prepubertal girls matched by age and 13 healthy pubertal girls matched by pubertal stage. For this purpose, the comparisons were performed between pre- and pubertal controls to identify changes in normal pubertal transition and CPP versus pre- and pubertal controls.

**Results:**

Analysis of methylation changes associated with normal pubertal transition identified 1006 differentially methylated CpG sites, 86% of them were found to be hypermethylated in prepubertal controls. Some of these CpG sites reside in genes associated with the age of menarche or transcription factors involved in the process of pubertal development. Analysis of methylome profiles in CPP patients showed 65% and 55% hypomethylated CpG sites compared with prepubertal and pubertal controls, respectively. In addition, interestingly, our results revealed the presence of 43 differentially methylated genes coding for zinc finger (ZNF) proteins. Gene ontology and IPA analysis performed in the three groups studied revealed significant enrichment of them in some pathways related to neuronal communication (semaphorin and gustation pathways), estrogens action, some cancers (particularly breast and ovarian) or metabolism (particularly sirtuin).

**Conclusions:**

The different methylation profiles of girls with normal and precocious puberty indicate that regulation of the pubertal process in humans is associated with specific epigenetic changes. Differentially methylated genes include ZNF genes that may play a role in developmental control. In addition, our data highlight changes in the methylation status of genes involved in signaling pathways that determine the migration and function of GnRH neurons and the onset of metabolic and neoplastic diseases that may be associated with CPP in later life.

**Supplementary Information:**

The online version contains supplementary material available at 10.1186/s13148-024-01683-1.

## Background

Puberty is a crucial biological process finalized to achieve full reproductive capacity [1]. This phase begins with an increase in pulsatile GnRH release that leads to the activation of complex signaling pathways in the hypothalamic–pituitary–gonadal (HPG) axis, culminating in puberty onset [2].

The age of normal pubertal onset varies widely among girls, ranging from 8 to 13 years. The timing of this important phenomenon is strongly influenced by the genetic background, along with environmental factors such as nutrition, pollution and stress [3–6]. Recently, the COVID19 pandemic has been associated with an important increase in the incidence of female central precocious puberty all over the world, demonstrating the strong influence of the environment on pubertal timing [7].

Compelling evidence has documented that epigenetics provides an additional level of regulation to the mechanisms that regulate HPG axis activation. Ojeda's works have helped to elucidate the molecular basis of the epigenetic control of puberty by identifying the activity of two large families of transcriptional regulators, with repressive [such as polycomb (PcG)] or activating [such as thritorax (TrxG)] functions, that act through epigenetic mechanisms to control KISS1 gene expression, mainly in the ARC neurons, during the pubertal transition [8, 9]. However, the PcG complex is not the only repressor regulating centrally the puberty onset. It has been proposed that GATAD1, a member of the ZNF family of transcriptional repressors, directly represses human transcription of KISS1 (and TAC3) by recruiting histone demethylase (KDM1a), thereby reducing the activation of their promoters [10]. Moreover, whole-genome studies have documented an association between genetic variation denoted by single nucleotide polymorphisms, located near certain ZNF genes, and changes in the age of menarche in women, reinforcing the translational relevance of this epigenetic mechanism of pubertal onset control [11, 12].

Epigenetic repression mechanisms targeting genes required for the activation of GnRH neurons have recently been identified as a key component of the molecular mechanism underlying the central control of puberty. Previous animal studies on the GnRH neuron in the basal hypothalamus of rhesus monkeys revealed a connection between increased GnRH gene expression and decreased CpG methylation status during GnRH neuronal development [13].

Moreover, methylation of CpG islands is intricately involved in the regulation of imprinted genes, determining their expression patterns in a parent-of-origin-specific manner and playing a crucial role in various developmental processes. Several imprinted genes were associated with the age of menarche in a large cohort of European women [14]. In particular, loss-of-function mutations in the paternally expressed imprinted genes makorin ring finger 3 (MKRN3) and delta-like 1 homolog (DLK1) have been shown to cause central precocious puberty, suggesting the important role of imprinted genes in regulating puberty timing [15–18].

In humans, global methylation changes have been demonstrated during adolescence [19], both in females and males [20, 21], further supporting the evidence that epigenetic modifications might be associated with pubertal development.

On the other hand, the timing of puberty onset and the hormonal and metabolic changes occurring during development contribute to susceptibility to a wide range of diseases later in life, like obesity, diabetes, PCOS and some types of neoplasms [22–25]. In particular, the earlier age of onset of puberty in females may have a negative impact on their future life and epigenetic changes may also contribute to this phenomenon.

To the best of our knowledge, scientific literature on global DNA methylation in patients with disrupted puberty onset (i.e., CPP) is scarce [26].

In the present study, we analyzed DNA methylation profiles in peripheral blood leukocytes in a cohort of Caucasian girls with idiopathic central precocious puberty, comparing them to healthy girls matched for age and pubertal stage; moreover, we examined changes in normal puberty by comparing healthy girls in prepubertal and pubertal stages.

## Results

### Population

Clinical and laboratory characteristics of the entire cohort are shown in Table 1. The prepubertal control girls (CTPP) had a mean age of 7.78 ± 1.01 years, they were all at Tanner stage I, while the pubertal control girls (CTP) had a mean age of 13.49 ± 1.48 years and at least a pubertal stage III with reported onset of puberty 12 months before the visit. No difference in BMI SDS existed between CPP patients and pubertal controls (0.58 ± 0.96 and 0.32 ± 0.98, respectively; *p* = 0.62). Neither difference between CPP patients and prepubertal controls was found in terms of BMI SDS (0.58 ± 0.96 and 0.87 ± 1.25, respectively; *p* = 0.45).

**Table 1:** Clinical and Laboratory characteristics of patients with CPP (central precocious puberty) and controls groups (CTPP and CTP)

	CPP	CTPP	CTP	*p* value CPP versus CTPP	*P* value CPP versus CTP
Age, years	7.36 ± 0.85	7.78 ± 1.01	13.49 ± 1.48	0.841	> 0.001
Δ EO-EC	1.69 ± 1	N.A	N.A		
Uterus Longitudinal diameter, cm	40 ± 6.9	N.A	N.A		
BMI, SDS	0.58 ± 0.96	0.87 ± 1.25	0.32 ± 0.98	0.457	0.517
Tanner Stage	2 (2–3)	1	3 (2–4)	< 0.001	0.06
LH, IU/L	1.3 ± 0.92	N.A	3.36 ± 1.81*		
FSH; IU/L	5.6 ± 2.22**	N.A	5.36 ± 2.50*		0.928
Peak-LH, IU/L	6.7 ± 1.6**	N.A	N.A		0.938
17β-estradiol, pg/mL	26.5 ± 10.3	N.A	33.8 ± 22.77*		0.264

The parameters are expressed as mean ± standard deviation, except for Tanner stage reported as median (min and max value). **Peak-LH and peak-FSH have been measured only in five patients, whose basal hormonal levels were not diagnostic for CPP.*Mean data are available in a subgroup of 10 patients. *p* value was calculated for CPP versus CTPP group and CPP versus CTP group

### Differentially methylated genomic regions during normal and early puberty

The examination of DNA methylation changes during puberty involved firstly the assessment of differentially methylated region (DMR) in prepubertal (CTPP) versus pubertal controls (CTP) revealing the presence of 32 DMRs (with an FDR < 0.05 and |Δ*B*|> 5%), with the 59% becoming more methylated at puberty (Additional file 1, Table 1). These 32 DMRs harbored the promoter region of genes involved in immune and inflammatory pathways, as the TNF promoter (Additional file 2). In addition, we applied epigenetic Landscape In Silico deletion Analysis (LISA) to search for enriched transcription factors that are potentially regulating genes associated with our DMRs. Among the first twenty significant ones (Additional file 3), we found the estrogen (ESR1) and progesterone (PR) receptor genes, involved in the action of sex hormones, and DNMT1 involved in the maintenance of DNA methylation processes affecting gene expression.

Comparison between CPP girls and prepubertal controls (CTPP) reported only nine DMRs (five hypomethylated and four hypermethylated) (Additional file 1, Table 2). Given the small number of identified DMRs, the IPA analysis found few pathways, some involved in neuronal differentiation and signaling, such as Semaphorin and GABA receptor signaling (Additional file 2). The search for transcription factors produced no significant results for this comparison (Additional file 3).

**Table 2:** DMR related to physiological pubertal transition in this study that were previously reported by Almstrup

Pre- and post-pubertal control groups							
DMR ID	CHR	START	END	Number of CpGs	FDR *p* value	Mean beta difference	Gene name
DMR_6	chr3	149094653	149096029	10	0.00236454	0.101705106	TM4SF1
DMR_122	chr9	125795283	125795935	7	0.02860196	0.094379721	GPR21
DMR_121	chr9	130524573	130525059	8	0.0282297	0.092866176	SH2D3C
DMR_62	chr8	38831148	38831857	10	0.01271887	0.091087731	HTRA4
DMR_38	chr2	33359198	33359688	9	0.00939611	0.090245406	LTBP1
DMR_46	chr8	27468684	27469338	8	0.01326348	0.089254	CLU
DMR_94	chr20	57582706	57583091	9	0.02048807	0.080482792	CTSZ
DMR_39	chr6	10520809	10521715	8	0.0124776	0.075281035	GCNT2
DMR_100	chr11	47399813	47400330	10	0.01943334	0.062760412	SPI1
DMR_169	chr16	89043171	89043707	8	0.03546119	0.055000527	CBFA2T3
DMR_143	chr6	31543300	31543686	8	0.03182821	0.053540566	TNF*
DMR_67	chr15	91427361	91428456	11	0.01540742	0.053309161	FES
DMR_115	chr10	71892694	71893159	9	0.02349373	0.049537353	AIFM2
DMR_148	chr12	6492890	6493521	6	0.03723976	0.047001034	LTBR
DMR_107	chr22	26875404	26876075	10	0.02232869	0.038810882	HPS4
DMR_173	chr7	8302094	8302301	6	0.04949676	0.034295838	ICA1
DMR_137	chr11	2321770	2321955	7	0.03766717	0.031967415	C11orf21
DMR_165	chr16	70323463	70323915	9	0.02902937	− 0.006027156	DDX19B
DMR_12	chr4	15780728	15781202	3	0.04472632	− 0.009104024	CD38
DMR_15	chr11	88071152	88071458	3	0.04620157	− 0.013501212	CTSC
DMR_91	chr11	45167702	45168039	5	0.04064525	− 0.023107519	PRDM11
DMR_159	chr12	107974444	107974897	6	0.04519509	− 0.047282953	BTBD11
DMR_30	chr3	52321636	52321767	4	0.04358886	− 0.050656554	GLYCTK
DMR_176	chr1	25291695	25292412	8	0.03814284	− 0.056905781	RUNX3
DMR_145	chr12	9821504	9822287	7	0.04067972	− 0.070023733	CLEC2D
DMR_19	chr6	31539539	31540456	15	0.00598373	− 0.078845575	LTA

Likewise, the results obtained from CPP and pubertal healthy controls (CTP) revealed 47 DMRs of which 78% were hypomethylated in the CPP group (Additional file 1, Table 3). Among the ten most significant regions were those containing the promoter of the KCNAB3 and LTA genes. In addition, also the region near MKRN3, one of the most important genes in pubertal development, is less methylated in girls with central precocious puberty. (Additional file 2) The search for transcription factors for this comparison also revealed the presence of target genes transcribing for hormone receptors in the top twenty list (Additional file 3).

### Distribution of genomic characteristics of differentiated CpG sites

**Fig. 1:**
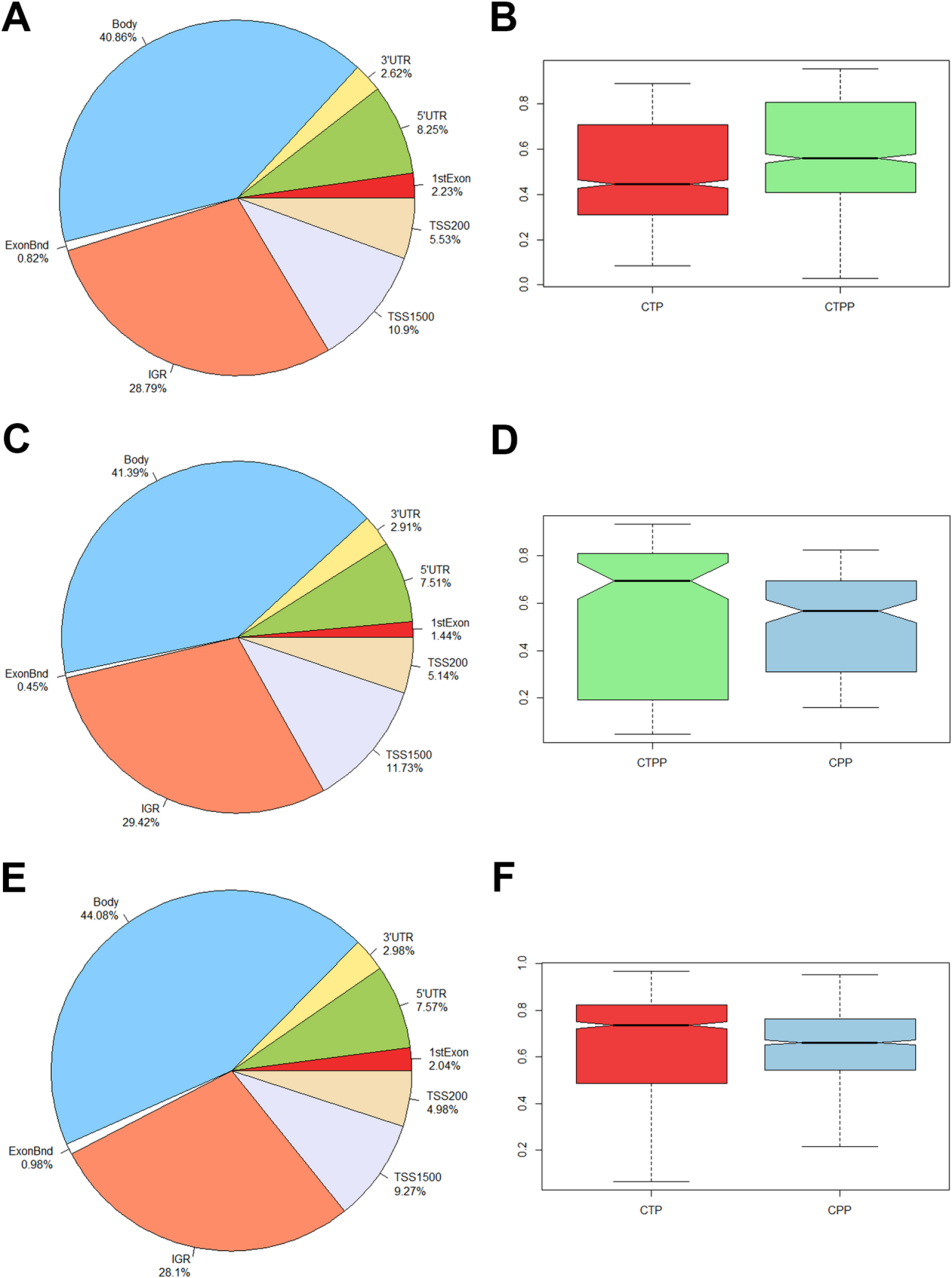
Distribution of the differentially methylated CpG sites identified for each comparison along the several genomic features. On the left (**A**: CTP vs. CTPP, **C**: CTPP vs. CPP, **E**: CTP vs. CPP), pie charts showing the percentage of DMPs for each genomic localization. On the right (**B**, **D**, **F**), boxplots showing the beta-values distribution per group (red for CTP, light blue for CPP and green for CTPP)

Given the small number of DMRs identified, we explored methylation levels at isolated CpG sites in more detail. In the comparison between prepubertal (CTPP) and pubertal controls (CTP), we observed that a significant proportion of the differentially methylated CpG positions (DMPs) (FDR < 0.05 and |Δ*B*|> 10%) were situated in intergenic regions (IGR) and within the body of genes (Body). Notably, approximately 25% of these DMPs were found in promoter regions, comprising TSS1500 (10.9%), TSS200 (5.53%), 5’UTR (8.25%) and 1st exon (2.23%) (Fig. 1A). Interestingly, a global analysis revealed that all the DMPs exhibited a higher level of methylation in CTPP compared to CTP (Fig. 1B). To assess changes in methylation linked to early puberty, individuals with central precocious puberty (CPP) were compared with healthy controls matched for age (CTPP) and pubertal stage (CTP). Also, the comparison between CPP subjects and prepubertal healthy controls (CTPP) showed the largest percentage of DMPs in the IGR (29.42%) and body (41.39%) of the genes, while only a small percentage in the transcription start sites and on the promoter of the neighboring regions of the genes (TSS1500 11.73%, TSS200 5.14%, 5'UTR 7.51% and 1st exon 1.44%) (Fig. 1C). Generally, all the differentially methylated CpG sites exhibit a pattern of hypomethylation in CPP (Fig. 1D). The distribution of the genomic features in the CPP versus CTP group reports the same trend found in previous comparison with a greater presence of DMPs in the IGR and body of genes and a pattern of hypomethylation in the CPP group (Fig. 1E–F).

### Changes in DNA methylation associated to the pubertal stage

**Fig. 2:**
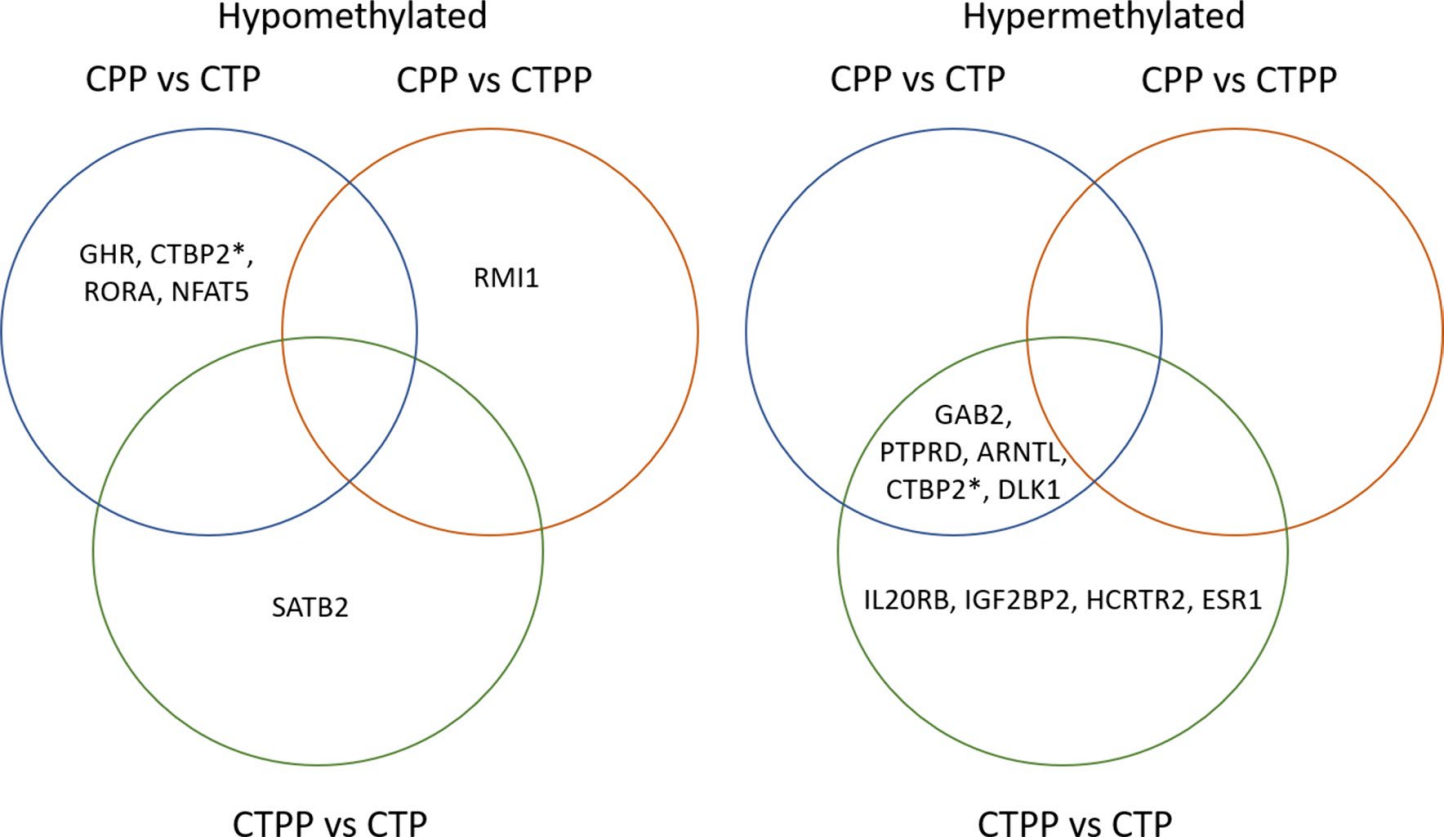
Venn diagrams showing the methylation status of genes associated to the age of menarche. The intersection regions show the differentially methylated genes in common with two or more groups. *CTBP2 reported a hypomethylated CpG in the 5'UTR region and a hypermethylated CpG at TSS1500

**Fig. 3:**
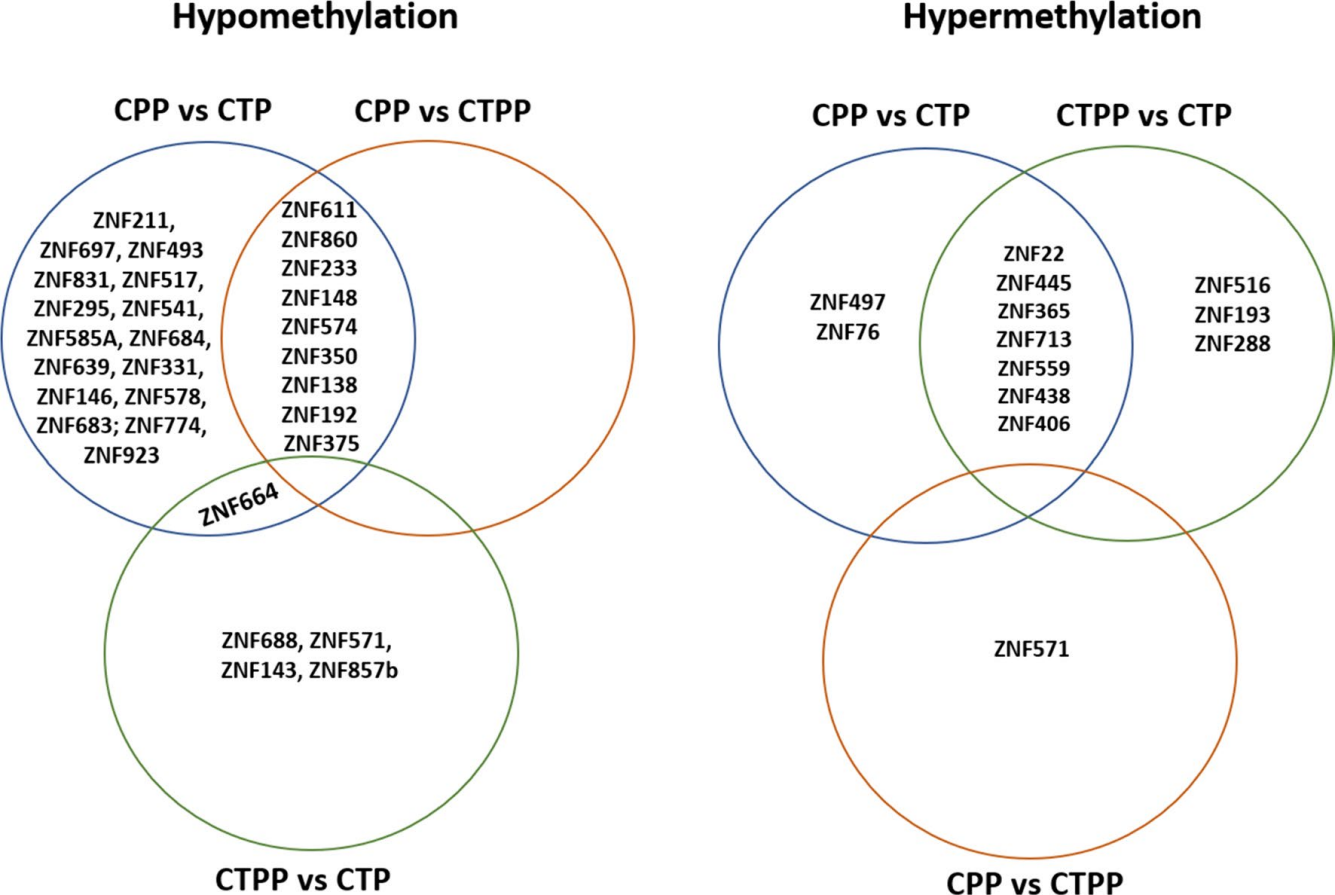
Venn diagrams showing methylation status of ZNF genes. The intersection regions show the differentially methylated ZNFs in common with two or more groups

Methylation studies often detect a predominance of differentially methylated sites in regulatory regions attributing a strong significance on gene expression while the role of differentially methylated CpGs in regions distant from the promoter is still uncertain. For this reason, in our study, we decided to consider only CpGs that are mapped on transcription start sites or promoter regions that, theoretically, could influence the regulation of the respective genes. Based on this, the comparison between prepubertal (CTPP) and pubertal healthy girls (CTP) revealed the presence of 1006 differentially methylated CpG sites with the majority of them (863 DMPs, 85.78%) being hypermethylated in prepubertal samples (Additional File 4). The association of these DMPs with the nearest gene revealed some genes that have been previously associated with age of menarche (Fig. 2) (Additional file 5, Fig. 1): SATB2, PTPRD, ARNTL, GAB2, CTBP2, IL20RB, IGF2BP2, HCRTR2, ESR1 [11] and DLK1 [16, 27]. Interestingly, 15 genes harbor differentially methylated CpG sites encoded for ZNFs (Fig. 3).

**Fig. 4:**
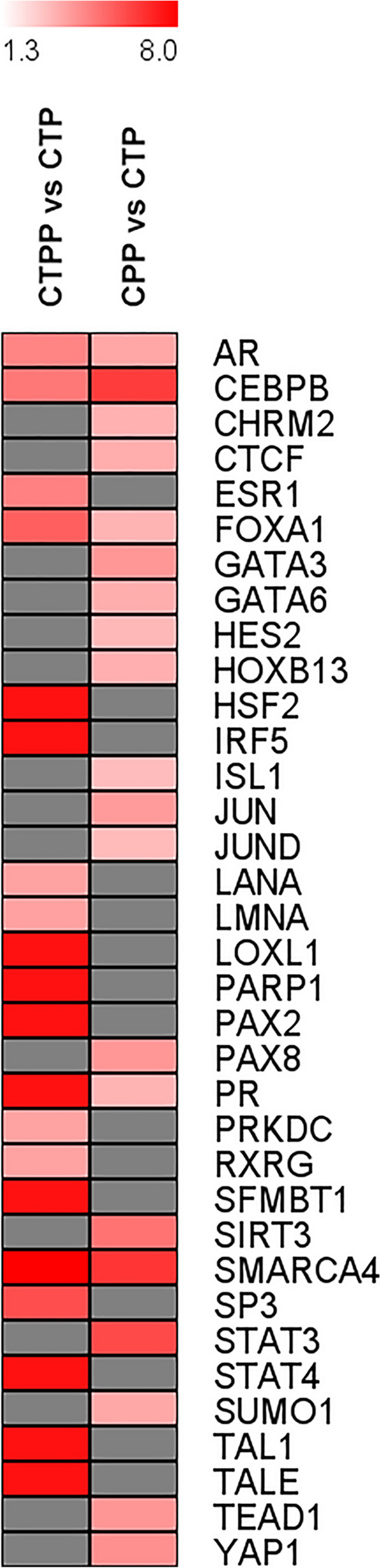
Heatmap showing the top twenty relevant transcription factors identified as target of the differentially methylated CpG sites detected. Colors from light red to dark red represent the increasing log FDR associated with that transcription factor. The grey boxes are TFs not present in the specific comparison

Subsequently, considering the list of genes with differentially methylated promoters, we performed a detailed bioinformatics analysis, to search  for putative transcription factors (TF) which are potentially regulating genes associated with our DMPs. In this way, we detected 189 TFs (FDR < 0.05). The top 20, shown in Fig. 4, that include ESR1, AR, PGR and SMARCA4, involved in sexual hormones action, ovarian carcinoma and in chromatin remodeling processes that influence gene expression. The full list of TFs is reported in Additional File 6.

**Fig. 5:**
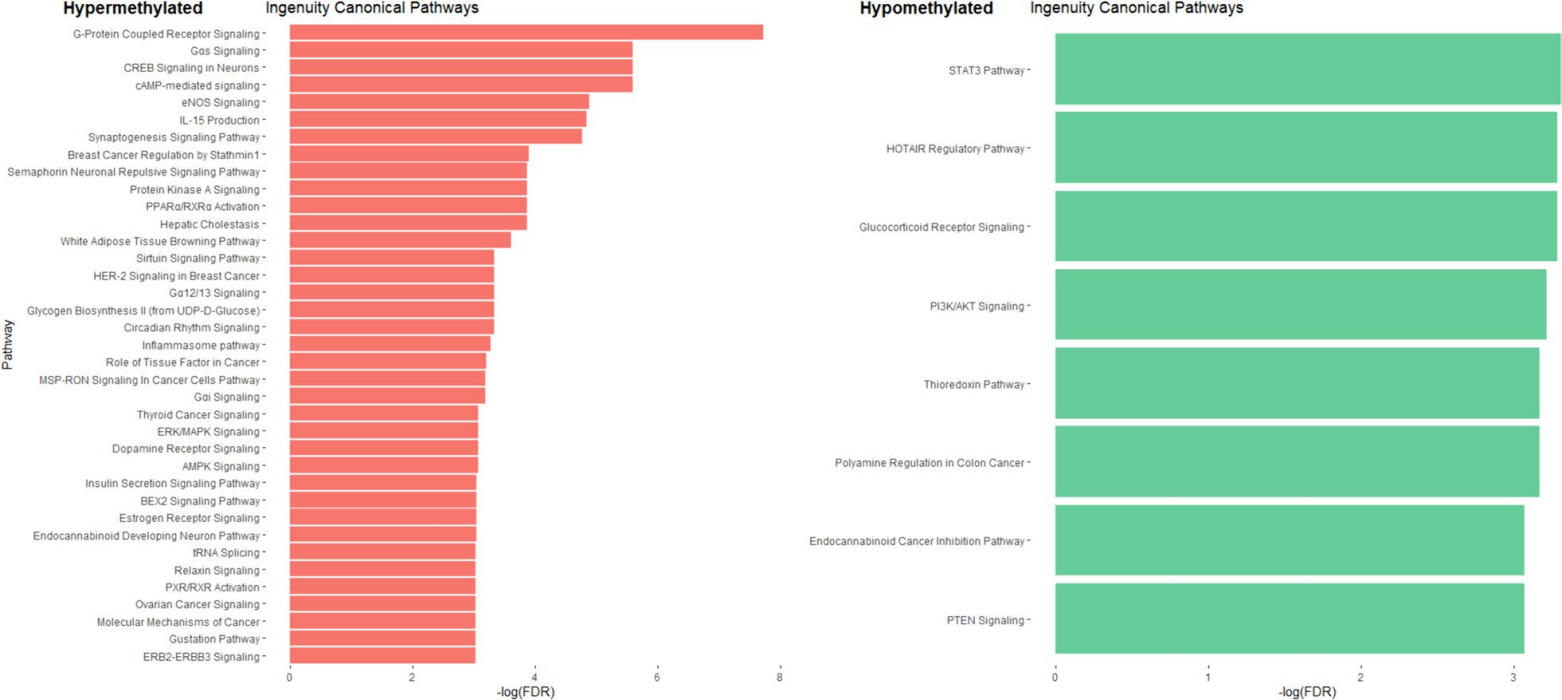
Histograms showing the Ingenuity canonical pathways identified comparing CTPP vs CTP. In red are represented the pathways where genes with hypermethylated CpGs were involved while, in green, those with hypomethylated ones. The figure shows only  part of total pathways

Moreover, among genes with differentially methylated promoters there was an enrichment of genes containing estrogen-responsive elements (EREs) (hypergeometric test with *p* value < 0.05), suggesting that some of the effects of estrogen signaling in puberty are modified through epigenetic mechanisms. However, when we could not find a correlation between beta-values and estrogen levels, no CpGs were found directly correlated with them (data not shown). Finally, to identify pathways that could be modulated by the differentially methylated genes, we performed Ingenuity pathway analysis (IPA). The results demonstrated how genes showing hypermethylated promoter are involved in pathways (in prepubertal patients) related to neuronal signaling (semaphorin and gustation pathways), estrogen signaling, cancer, in particular breast and ovarian cancer signaling, or metabolism (sirtuin signaling) (Fig. 5). The completed list of differentially methylated pathways is reported in Additional File 7.

### Changes in DNA methylation associated to central precocious puberty

**Fig. 6:**
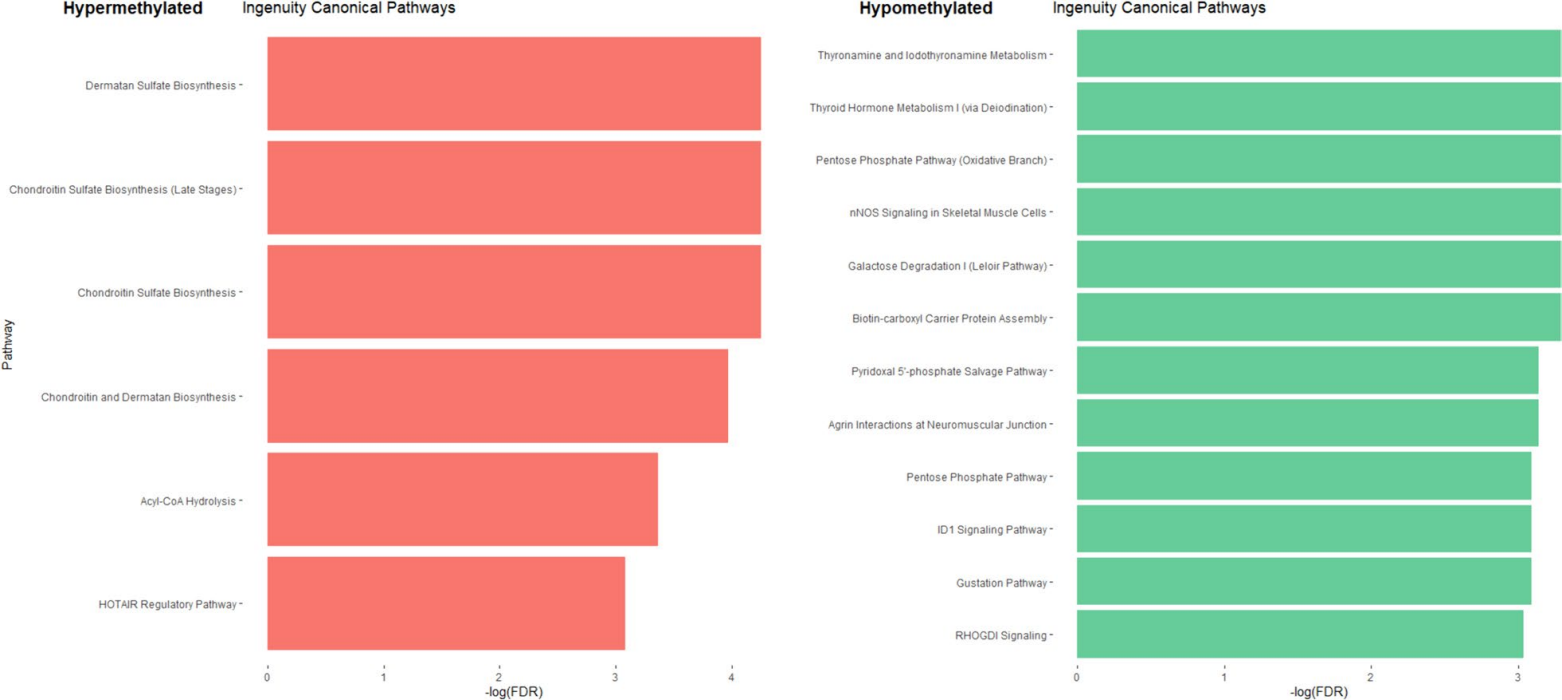
Histograms showing Ingenuity canonical pathways identified comparing CPP vs CTPP. In red are represented the pathways where genes with hypermethylated CpGs were involved while, in green, those with hypomethylated ones

Comparison between central precocious puberty (CPP) and prepubertal healthy girls (CTPP) revealed the presence of 156 differentially methylated CpG sites that were hypomethylated in the CPP group (102 DMP, 64.96%) (Additional File 8). Among the relevant genes, 10 differentially methylated ZNF were highlighted (Figs. 2, 3). As reported in Fig. 6, the Ingenuity pathway analysis (IPA) performed showed very few pathways with a methylation difference of more than 10%. The completed list of differentially methylated pathways is reported in Additional File 9.

Likewise, the analysis in CPP subjects and pubertal controls (CTP) showed a total of 1105 DMPs with 55.11% of DMPs hypomethylation (609 DMPs) in the CPP group (Additional File 10). The functional characterization of 1033 genes related to these 1105 DMPs showed the presence of 35 differentially methylated ZNFs and many genes associated with age of menarche, some of them (such as PTPRD, ARNTL, GAB2, CTBP2) resulted in common with the comparison prepubertal vs pubertal controls (Figs. 2, 3). Bioinformatic analysis of transcription factors detected 139 TFs (FDR *p-*value < 0.05) which represent the ones that are potentially regulating genes associated with our DMPs. The top 20 TFs are shown in Fig. 4 (full list in Additional File 6).

**Fig. 7:**
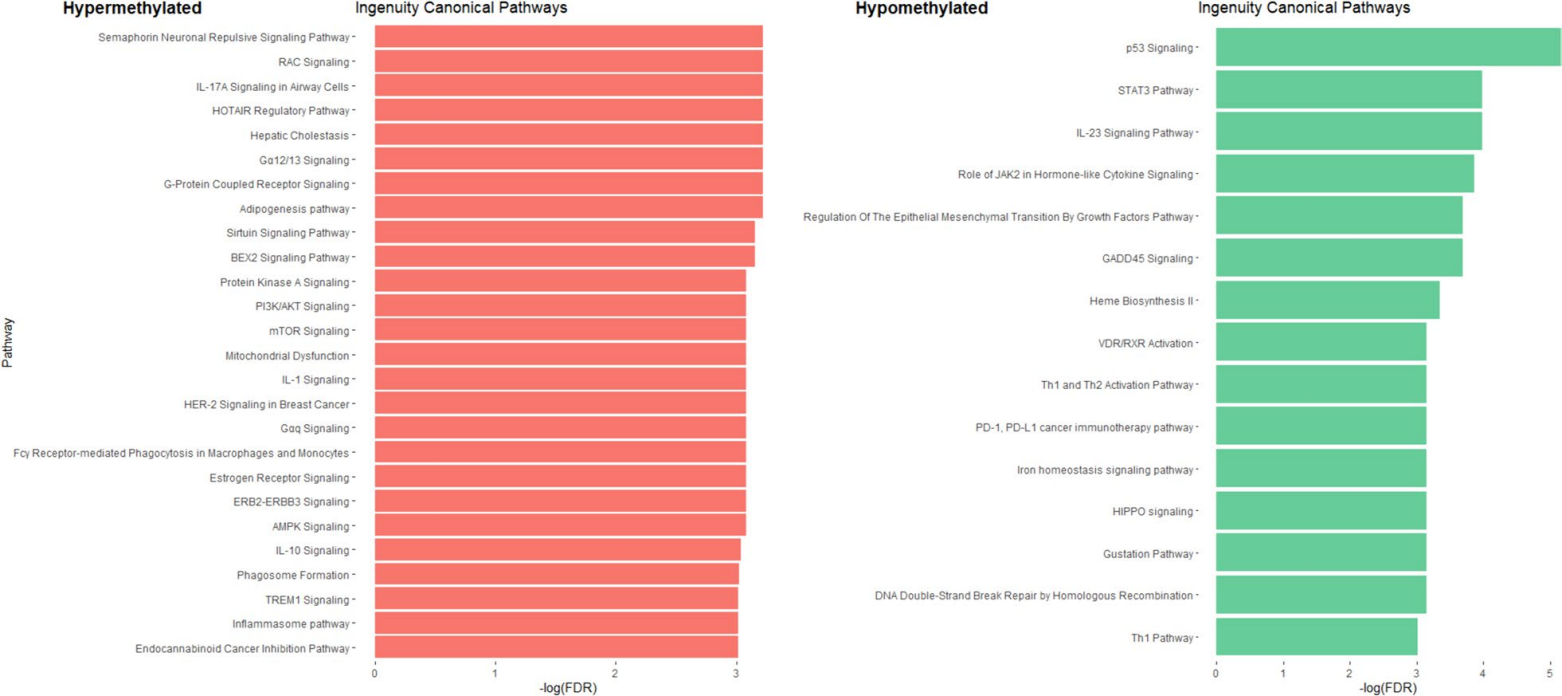
Ingenuity canonical pathway analysis between CPP and CTP. In red are represented the pathways enriched with hypermethylated CpGs while, in green, the hypomethylated ones. The figure shows only part of the total pathways

Ingenuity pathway analysis (IPA) showed an enrichment in signaling pathways such as neuronal signaling (semaphorin and gustation pathways), estrogen signaling, cancer and metabolism (sirtuin signaling and adipogenesis) (Fig. 7). The whole list of differentially methylated pathways is reported in Additional File 11.

### ZNF methylation

Given the potential contribution of ZNF genes to the pubertal process, we focused on the methylation signature of CpG falling in these genes. Our analysis revealed the presence of 43 differentially methylated ZNF genes, with 39.5% shared by at least two groups of subjects. The highest number of differentially methylated ZNFs was recorded for the CPP group, with 23 hypomethylated and nine hypermethylated ZNFs (Fig. 3). In particular, ZNF148 appears to positively regulate the GABA transporter promoter (SLC6A1) who acts as an inhibitor of GnRH secretion [28]; ZNF574 and ZNF350 are associated with ovarian cancer and breast cancer, respectively [29, 30]; ZNF138 is located in the region involved in determining Williams syndrome that is sometimes associated with CPP [31]; ZNF664 is involved in metabolic syndrome [32, 33]; ZNF445 is related to Temple syndrome and acts as a regulator of imprinting along with ZFP57 [34–37]; ZNF365 plays a role in regulating genomic stability [38].

## Discussion

In recent years, the understanding of epigenetic mechanisms underlying pubertal development has considerably accelerated. Around a decade ago, it was proposed an activation–inhibition mechanism that influences the transcriptional activity of GnRH neurons [39, 40]. An important component of the repressive arm of this mechanism has been identified in the Polycomb group (PcG) of transcriptional silencers which prevent the onset of puberty by silencing KISS1 gene in the arcuate nucleus (ARC) in the hypothalamus thus inhibiting the release of GnRH [8]. This effect is antagonized by the transcriptional activator group Trithorax (TrxG), which catalyzes histone modifications, such as H3K4 trimethylation and H3 acetylation, resulting in gene activation [8]. Despite this important discovery, the complexity and sex- and tissue-specific variability of these reversible changes have not yet completely clarified so far.

The aim of our project was to evaluate, through a minimally invasive method, such as blood sampling, the changes in genomic DNA methylation profiles during normal and precocious puberty.

Previous longitudinal studies have shown the dynamic nature of DNA methylation during pubertal development. Indeed, it has been reported that epigenetic modifications occur between the ages of 8 and 14 years with significant differences also related to gender [41, 42], suggesting that peripheral epigenetic changes can mirror biological processes related to puberty onset. A Danish study [41] has identified some differentially methylated regions that overlap with our data during the normal pubertal transition (Table 2). Similarly to our results, the region in proximity of LTA and TNF genes was reported by Almstrup to be among the regions with the greatest methylation difference across puberty development [41].

The small proportion of overlapped DMRs is not completely unexpected. The two studies differ in study design (longitudinal versus cross-sectional) and cohort composition, as Almstrup et al. [41] combined both sexes for analysis, whereas we only considered healthy girls or girls with CPP. Furthermore, genome-wide methylation patterns were assessed with two different methods, the Illumina 450K array and the MethylationEPIC BeadChips (850K). These systems differ not only for the number of probes but also for their proportions (Type I and Type II), and for the genomic regions tested. Furthermore, a decrease in correlation between the two methods using blood samples is known [43].

We also reported some regions with a different methylation status in the CPP group compared to controls of the same pubertal stage. Interestingly, the region in the proximity of KCNAB3, identified by Almstrup as hypermethylated at puberty, showed methylation with the same direction in our group of CPP patients, suggesting a possible, but still unclear, involvement in the pubertal process. Of particular interest is the different methylation that we reported in the region harboring MKRN3 in CPP compared to pubertal girls. Indeed, it has long been known that MKRN3 acts as a brake on puberty in mammals, probably by repressing the transcription of KISS1 and TAC3. An alteration in its methylation status could therefore be involved in an advancement of pubertal development.

However, given the few methylation differences reported for larger regions, we have focused on the individual differentially methylated CpGs. The analysis of DMPs identified a higher percentage of hypermethylated sites in the prepubertal phase, while slight hypomethylation was reported in subjects with CPP compared to healthy controls matched by age or pubertal stage. At puberty, demethylation of specific regions could trigger a number of key developmental mechanisms. The same trend was described by Thompson’s study in which approximately 55.3% of the differentially methylated probes (DMP) detected in females between 8 and 14 years were hypomethylated in the post-pubertal phase [42]. However, our analysis of genome-wide DNA methylation profiles does not allow for a causal inference, as differentially methylated CpGs can be both a cause and a consequence of pubertal advancement. Since methylation of a single CpG is unlikely to have a biological effect, such epigenetic modifications may nevertheless be useful markers for tracking pubertal transition. To the best of our knowledge, the only cross-sectional study on different methylation profiles in girls with normal and precocious puberty has been published by Bessa et al. [26]. In contrast to our results, Bessa showed higher DNA methylation in DMPs of subjects with CPP compared to pre- and pubertal controls, whereas methylation differences in the normal pubertal transition were only reported in the analysis of larger regions (DMR), with a hypermethylation trend recorded at the pubertal stage. Even in this case, the methods used (Human Methylation 450K vs Infinium MethylationEPIC BeadChips) and the cohorts, present considerable differences between the two studies, thus providing a bias that may affect the results. In fact, Bessa analyzed a small cohort of 10 Brazilian girls affected by familial forms of central precocious puberty, some of them with known mutations in the MKRN3 and DLK1 genes. The different ethnicity [44–46] and the possibility of a genetic cause of CPP given the strong familiarity could contribute to a different methylation profile that is therefore difficult to compare to the present study. However, an overlap of 32 DMPs was found with the data reported in CPP compared with pubertal subjects and 2 DMPs with the prepubertal, some showing methylation changes in the same direction (Additional file 5, Table 1). Furthermore, even if there are some discrepancies due to the methods used, we found a good degree of correlation between beta-values of CpGs in common between Bessa and our study (Additional file 5, Fig. 2).

Interestingly, many DM-CpGs detected in our study fall within the regulatory region of several genes (eight genes in CCP-CTP, ten in CTPP-CTP and one in CPP-CTPP) (Additional file 5, Fig. 1) associated with age of menarche in a previous GWAS analysis [11]. Even if there are many differences in the aim and the method of the two studies (i.e., probes of the methylation array are not suitable for studying SNPs and the SNPs IDs changed during the years), these data support the hypothesis of a possible role of these genes in the control of pubertal process and suggesting that DNA methylation can modulate their action in pubertal timing determination.

Several genes differentially methylated in our analysis are involved in important processes such as brain and synapse development (i.e., SATB2 and CTBP2), cell growth and differentiation (i.e., PTPRD), inflammation and the immune system (i.e., IL20RB) or metabolism (i.e., IGF2BP2). In addition, also the DLK1 gene showed a state of hypermethylation in CPP subjects. According to the literature, DLK1 plays an inhibitory role in the regulation of puberty, as its deficiency has been associated with a CPP phenotype in both non-syndromic (mutation with loss of function) [18] and syndromic conditions, such as Temple syndrome, due to altered methylation on chromosome 14q32, where the gene is mapped.

Interestingly, some of our differentially methylated genes are involved in the regulation of sleep–wake rhythm (HCRTR2), or controlling circadian rhythm oscillations (ARNTL), known to be among the stimuli that can affect the timing and the progression of puberty [39, 47].

In humans, GWAS studies have also revealed associations between age at menarche and SNPs located near Zinc finger (ZNF) genes, suggesting that they may influence human pubertal development [10]. In fact, recently, it was discovered that among the transcriptional targets of ZNF483 there are many genes associated with the age of menarche and that an increased binding of ZNF483 to them confers a precocious development [10]. This was further confirmed by functional domain-specific gene burden analyses, which showed that mutations in ZNF483, particularly within the zinc finger domains, confer delayed puberty by disrupting the protein's ability to bind its multiple DNA targets [48]. In addition, MKRN3, also known as ZNF127, has been shown to inhibit the human pubertal onset and its loss-of-function mutations are the most frequent cause of familial CPP [17, 49, 50].

Many of these ZNFs appeared to be associated with processes involved in development or diseases/conditions related to precocious puberty in previous studies. For instance, ZNF148 indirectly acts on GnRH secretion, regulating the GABA transporter promoter (SLC6A1) [51].

Of particular interest is the hypermethylation that we found in the ZNF455 gene. The study by Takahashi et al. revealed an important role of ZNF445 in the regulation of imprinting together with ZFP57, the only zinc finger found hypomethylated in pubertal controls in the study by Bessa et al. [26]. In mice, ZFP57 plays the predominant role in the maintenance of imprinting, whereas, in its absence, ZFP445 is required to preserve methylation in a subset of imprinting control regions such as those present in the MEG3/DLK1:IG-DMR, that involved in Temples’ syndrome. In humans, the mild effects of ZFP57 mutations on imprinting support a less important role of this protein in the maintenance of imprinting, while the expression profile of ZNF445, its intolerance to loss-of-function mutations and the ability of its product to bind and install heterochromatin to imprinting central regions, strongly suggest that ZNF445 is an important factor in the maintenance of early embryonic imprinting [37]. Indeed, the discovery of a pathogenic variant of ZNF445 responsible for Temple syndrome and multi-locus imprinting disorders confirms its role in imprinting mechanisms [35]. The hypermethylation of ZNF445, evidenced by our analysis in subjects with CPP, compared to both control groups, confirms its possible involvement in pubertal development. Further studies on ZNF455 are required to clarify its function on puberty timing control.

The search for transcription factors targeting DM-CpGs revealed higher scores for hormone receptors such as progesterone, androgens and estrogens, suggesting that epigenetics may modulate hormone action and response. Although the correlation analysis between estrogen levels and DM-CpG in our patients did not produce any results, many DMPs were located within or near genes containing high-affinity estrogen-responsive elements that are implicated in the pubertal timing and endocrine system development thus confirming previous findings of longitudinal studies on normal pubertal transition [41, 42]. The lack of correlation could depend on the cross-sectional setting of our study and the similarity in the hormone levels in our patients, which does not let us to detect differences between the groups. Moreover, in the present cohort, estrogen levels are available only for CPP and pubertal patients, while in prepubertal patients, they were not measured (moreover estrogens are often not detectable in prepubertal subjects). This limitation thus does not allow to establish a causal relationship between estrogen levels and epigenetic changes. However, IPA analysis showed that estrogen signaling pathways and pathways related to estrogen-dependent cancers in women, in particular breast and ovarian cancer, are differently methylated in our cohort; this finding could represent a link between some estrogen-dependent conditions and early menarche [52]. A recent study in young Finnish adults also reported a correlation between methylation of some CpG sites and breast, ovarian and endometrial cancer in the models analyzed [53]. Indeed, during puberty, woman's breast and reproductive system are in a window of vulnerability to external factors that can promote molecular damage acting in different ways; methylation could be one of these mechanisms. In addition to the critical role that estrogens play in female puberty, they also modulate inflammation and immune responses [54, 55], both signaling pathways differently methylated in the prepubertal and CPP groups in our study.

In addition, our results also showed an enrichment of differentially methylated genes involved in key developmental pathways, such as neuronal migration, immune establishment and metabolism, whose differential methylation could therefore suggest their involvement in determining diseases, such as cardiovascular and metabolic disorders, that may be associated with early puberty. These signaling pathways include sirtuins, which play an important role in energy balance and energy sensing at the central level. Indeed, recent evidence demonstrated their role not only in neuronal migration but also in the metabolic control of pubertal development [56, 57].

Other interesting differently methylated pathways are semaphorin and gustation ones. It has been shown that intercellular communication within the neural GnRH network is mediated in part by semaphorin signaling, which plays a key role in the development of hypothalamic circuits but also in the control of GnRH release by circulating sex steroids [58]. Insufficient semaphorin signaling contributes to some forms of reproductive disorders in humans, such as hypogonadotropic hypogonadism [59], defective neuroendocrine control of the adult ovarian cycle [60] and obesity [61]. Among the different genes operating in this pathway, we point out SEMA6A that has been shown to control several biological processes including cytoskeleton remodeling, cell proliferation and survival [62, 63]. Characterization of SEMA6A by in silico, in vivo and in vitro analysis, and identification of deleterious genetic variants of semaphorin in patients with delayed pubertal onset confirmed the involvement of SEMA6a in the development of GnRH neurons and the onset of puberty [64].

Moreover, we reported that also the G-protein-coupled receptor (GPCR) signaling pathway appears to be differentially methylated between pre- and pubertal subjects as well between CPP and pubertal controls. GPCRs play particularly important roles in the neurosensory and endocrine systems. The hypothalamic-pituitary–gonadal (HPG) axis in humans comprises at least six GPCRs whose genetic defects, alone or in combination with other gene variants, lead to puberty disorders [65]. Although alterations in the methylation of GPCR-associated signaling have been reported so far mainly in the development of certain types of tumors [66–68], epigenetic modifications of this pathway could also be linked to normal or early pubertal development [69]. One example is the different methylation, found during the normal pubertal transition, of the relaxin signaling pathway, which, acting through GPCRs, seems to be closely related to connective tissue remodeling, which affects the female reproductive system by promoting the growth of the cervix, uterus and mammary gland [70].

Although the present analysis of methylation at puberty provides important findings, it certainly has limitations. The first one is the lack of RNA samples to test correlations between different degrees of DNA methylation and expression of relevant genes. Secondly, the use of nucleated blood cells and not of a specific tissue is a limitation. Tissues involved in pubertal development, such as the hypothalamus and gonads, are difficult to obtain in humans for both ethical reasons and sampling difficulties. However, many studies have reported the same methylation trend and gene expression in both blood and other tissues in mice and humans [71], confirming that blood is a valid surrogate for assessing epigenetic mechanisms during the pubertal transition. Furthermore, we realized that, even if the possibility to correlate our analysis with other dataset (such as ChIP-Seq or RNA-Seq) could be a huge improvement in this topic, unfortunately, to date, it is arduous to find such data on CPP patients.

In addition, one must also consider the effect of many exogenous factors, such as diet, environmental factors or endocrine disruptors, which may in turn anticipate pubertal development and also cause changes in methylation, increasing inter-sample bias and complicating data interpretation.

In conclusion, our study provides significant evidence that during both normal and precocious puberty, DNA methylation changes occur. Both normal pubertal subjects and CPP patients have a trend in hypomethylation but the regions involved are different, suggesting at least partially different underlying mechanisms.

Many of the differentially methylated signaling pathways are involved in physiological processes, such as the migration and function of GnRH neurons, development and growth, or pathological processes, such as the onset of metabolic and neoplastic diseases that may be associated with CPP in later life. Furthermore, the over-representation of ZNF among differentially methylated genes probably suggests a role in developmental control.

## Conclusions

By demonstrating a different pattern of DNA methylation in girls with normal and precocious puberty, we suggest that epigenetic mechanisms are relevant to pubertal timing in humans.

These differences in methylation patterns involve genes associated with age at menarche, ZNFs and are enriched in pathways involved in metabolism, neuron migration and some cancers, thus suggesting that DNA methylation could be a link between pubertal development and some puberty-related diseases later in life. Studies on epigenetics offer correlations but not necessarily causation. This means that we cannot establish whether DNA methylation is the cause or the consequence of the onset of the pubertal process. Future longitudinal studies on DNA methylation during normal and disrupted pubertal development will help clarify this complex relationship.

## Methods

### Populations

We enrolled a total of 46 female patients: 14 prepubertal controls (mean age 7.78 ± 1.01); 13 pubertal controls (mean age 13.49 ± 1.48) and 19 CPPs with a diagnosis of idiopathic CPP (mean age 7.36 ± 0.85) who were referred to Paediatric Endocrinology Units of the University of Campania “Luigi Vanvitelli,” Naples. Prepubertal and pubertal controls were selected from patients who came to the auxology outpatient clinic on suspicion of poor growth, which was subsequently ruled out, and were therefore considered healthy. Central hypothalamic–pituitary–gonadal activation was defined by measuring luteinizing hormone (LH) levels at baseline (> 0.3 mUI/mL) or a peak-LH > 5mUI/mL after GnRH stimulation test (0.1 mg Relefact LH-Releasing Hormone, Sanofi-Aventis, Frankfurt am Main, Germany). In all CPP patients, MKRN3 mutations were excluded. All patients underwent brain MRI showing normal hypothalamic-pituitary anatomy. Samples collection and genetic analysis were performed in the Department of Woman, Child, General, and Specialized Surgery of the University of Campania “Luigi Vanvitelli” Naples.

### Laboratory studies

All blood samples were drawn at 8:00 a.m. from an antecubital vein, clotted, centrifuged, and serum was stored at − 20 °C until analyses were performed. FSH and LH concentrations were determined by immunochemiluminometric assay (ICMA) with inter-assay coefficient of variation less than 5%. Serum 17-β-estradiol was measured by using competitive chemiluminescent immunoassay using coated magnetic particles with inter-assay coefficient of variation less than 5%.

### Genetic studies

DNA was extracted from peripheral blood samples using the Wizard Genomic DNA Purification Kit (Promega, Madison, WI, USA) following the manufacturer's instructions. By Sanger sequencing, all patients with precocious puberty were screened for the MKRN3 gene to exclude significant mutations in the coding region.

### Samples preparation and genome-wide DNA methylation analysis

Genomic DNA was extracted from peripheral blood leukocytes using standard procedures. DNA quality and quantity were assessed by NanoDrop (Thermo Fisher Scientific) and electrophoresis on 1% agarose gel. Microarray analyses were performed by Genomix4life S.R.L. (Baronissi, Salerno, Italy). The DNA concentration in each sample was assayed with a Qubit fluorometer. Bisulfite converted DNA (250 ng) was used for the analysis of whole-genome methylation using the MethylationEPIC BeadChip (Illumina, San Diego, CA, USA). In brief, bisulfite converted DNA was whole-genome amplified for 20 h followed by end-point fragmentation. Fragmented DNA was precipitated, denatured and hybridized to the BeadChips for 20 h at 48 °C. The BeadChips were washed and the hybridized primers were extended and labeled before scanning the BeadChips using the Illumina iScan system. Data analysis was performed as described in Casarotto et al. 2022 [72]. CpGs beta-values were obtained using ChAMP [73] on R (v. 4.2.0). Values of blood cell composition were obtained from the patients and missing values were imputed with a lasso linear regression model. Singular value decomposition was not able to detect the blood cell composition as factor that accounts for a substantial fraction of variation, for this reason, no corrections were performed. Only CpGs with a detection *p*-value < 0.01 have been considered for further analysis. CpGs were considered differentially methylated if the beta difference between the groups (Δ*β*) was more than 10% (|Δ*β*|> 0.1) and FDR values < 0.05. Further analyses were performed on the CpGs located within promoter regions, which are defined as CpGs annotated in the TSS1500, TSS200, 1 Exone and 5’UTR. DMR analysis was performed with ChAMP using the BumpHunter model with default parameters. DMR with FDR values > 0.05 and |Δ*β*|< 0.05 were excluded. The canonical pathways were generated through the use of QIAGEN IPA (QIAGEN Inc., https://digitalinsights.qiagen.com/IPA) and only the ones with -log(p-value) > 1.3 were considered significant. A list of regions with high-affinity EREs in the human genome was downloaded from the “MOUSE AND HUMAN ERE DATABASES” (http://mapageweb.umontreal.ca/maders/eredatabase/) [74] and intersected with the genomic coordinates of the differentially methylated positions (DMPs). Hypergeometric test with *p*-value < 0.05 was used to assess the enrichment of ERE in our DMPs. Transcription factors were found using “epigenetic Landscape In Silico deletion Analysis” (LISA—http://lisa.cistrome.org/) [75] with default parameters. All the statistics, pie charts and heatmaps were obtained using R.

### Supplementary Information

**Additional file 1**: complete list of differentially methylated region (DMR) with | Δβ| > 0.05. The file is divided in three table, one for each comparison.

**Additional file 2**: significant networks of genes (from Ingenuity pathway analysis) associated with the analysis of DMRs. The file is divided in three table, one for each comparison.

**Additional file 3**: list of transcription factors. The file is divided in three table, one for each comparison.

**Additional file 4**: complete list of differentially methylated CpG sites with | Δβ| > 0.1. The file is divided in up- and down-methylated CpG for the CTPP vs CTP comparison.

**Additional file 5**: comparison with Bessa and Perry studies.

**Additional file 6**: list of transcription factors for the comparison between CTPP vs CTP groups and CPP vs CTP groups.

**Additional file 7**: Significant networks of genes (from Ingenuity pathway analysis) associated with the analysis of DMPs detected in the comparison between CTPP vs CTP groups.

**Additional file 8**: complete list of differentially methylated CpG sites with | Δβ| > 0.1. The file is divided in up- and down-methylated CpG for the CTPP vs CTP comparison.

**Additional file 9**: Significant networks of genes (from Ingenuity pathway analysis) associated with the analysis of DMPs detected in the comparison between CPP and CTPP groups .

**Additional file 10**: complete list of differentially methylated CpG sites with | Δβ| > 0.1. The file is divided in up- and down-methylated CpG for the CTPP vs CTP comparison.

**Additional file 11**: Significant networks of genes (from Ingenuity pathway analysis) associated with the analysis of DMPs detected in the comparison between CPP and CTP groups.

## Data Availability

Raw methylation data and the normalized beta-values are available on ArrayExpress (E-MTAB-13950).

## Abbreviations

ARNTL: Aryl hydrocarbon receptor nuclear translocator-like
BMI: Body mass index
CPP: Central precocious puberty
CTBP2: C-terminal-binding protein 2
CTP: Pubertal control
CTPP: Prepubertal control
DLK1: Delta-like 1 homolog
DMP: Differentially methylated probe
ERE: Estrogen-responsive elements
ESR1: Estrogen receptor 1
GAB2: Grb2-associated binder2
GnRH: Gonadotropin-releasing hormone
GWAS: Genome-wide association study
HCRTR2: Hypocretin receptor 2
HPG: Hypothalamic–pituitary–gonadal
IGF2BP2: Insulin-like growth factor 2 MRNA binding protein 2
IL20RB: Interleukin 20 receptor subunit beta
KCNK9: Potassium two pore domain channel subfamily K member 9
KISS1: KiSS-1 metastasis suppressor
KCNAB3: Potassium voltage-gated channel subfamily A regulatory beta subunit 3
LTA: Lymphotoxin alpha
MKRN3: Makorin ring finger 3
PGR: Progesterone receptor
PTPRD: Protein tyrosine phosphatase receptor type D
SATB2: Special AT-rich sequence-binding protein 2
SEMA6A: Semaphorin 6A
SLC6A1: Solute carrier family 6 member 1
SMARCA4: SWI/SNF related, matrix associated, actin dependent regulator of chromatin, subfamily a, member 4
ZNF: Zinc finger
