# Supplementary material for: Methylome analysis in girls with idiopathic central precocious puberty
Source: Clin Epigenetics. 2024 Jun 22;16:82. doi: 10.1186/s13148-024-01683-1 (PMC11193236; doi:10.1186/s13148-024-01683-1)

Figure 1: Venn diagram show the number of genes differentially methylated in the present methylation analysis and the genes in which fall the SNPs identified by Perry et al.

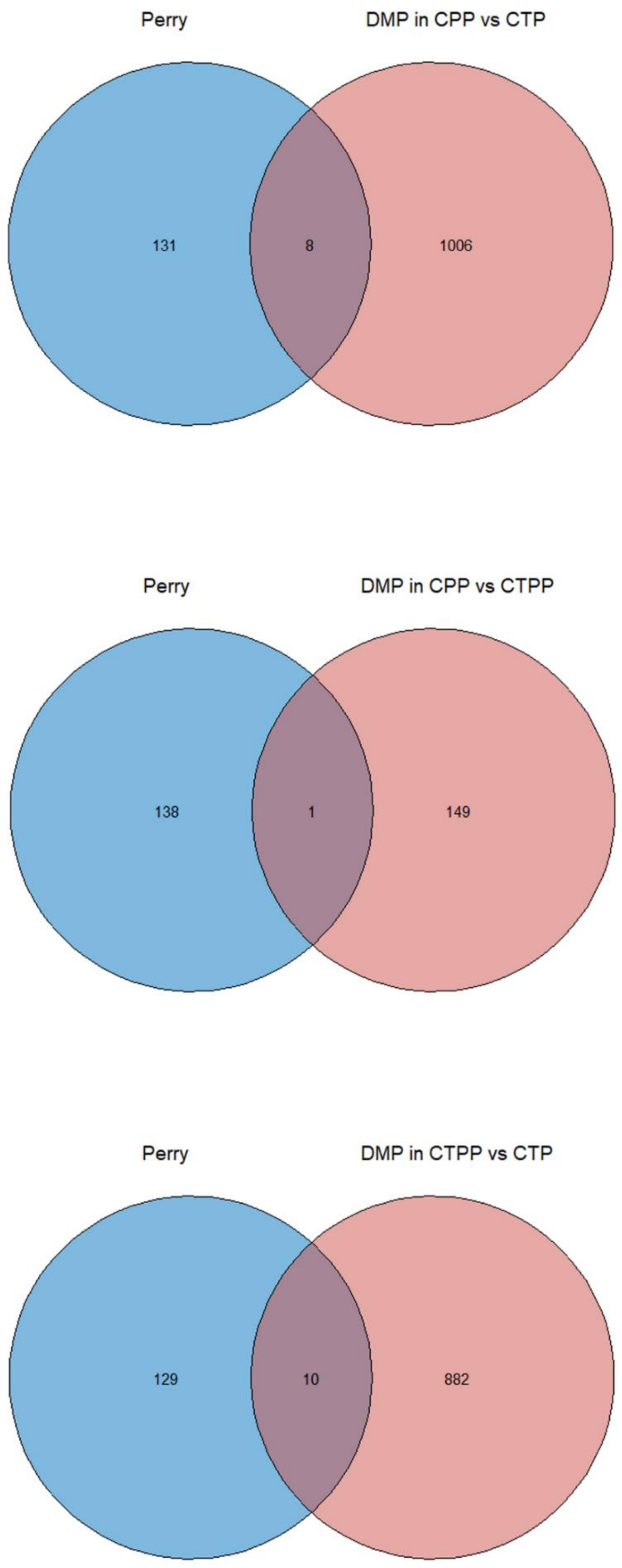

Table.1 DMPs related to CPP, pre- and post-pubertal control groups in this study that were previously identified by Bessa et al.

CPP and pubertal controls

CG_ID	CHR	adj.P.Val	deltaBeta	feature	cgi	gene
cg09463656	17	1.54E-10	0.173907234	TSS1500	shore	HS3ST3A1
cg25338843	17	2.01E-05	0.15284012	TSS200	shore	FAM171A2
cg24490154	X	5.33E-07	0.132127595	5'UTR	shelf	BCORL1
cg23546626	7	0.0005105	0.127576632	5'UTR	opensea	SLC37A3
cg26393261	6	3.97E-05	0.12712572	5'UTR	opensea	ATXN1
cg00978415	1	0.0010983	0.123812995	TSS1500	opensea	SELENBP1
cg22769890	10	0.0006997	0.121354301	TSS1500	shore	CTBP2
cg21898708	6	8.27E-10	0.120837134	TSS1500	shore	C6orf48
cg22095128	13	0.0029622	0.120189909	5'UTR	opensea	STARD13
cg27488007	10	0.0084404	0.118261561	5'UTR	shore	CTBP2
cg08122232	17	0.0003402	0.117752273	TSS1500	shore	EXOC7
cg24374538	19	1.06E-07	0.117100808	TSS1500	shore	RPS9
cg01349063	8	0.0010274	0.11423095	5'UTR	opensea	SNTG1
cg21334129	2	0.016538	0.113086786	5'UTR	opensea	CDC42EP3
cg02375313	6	0.0009503	0.111990423	5'UTR	shore	EXOC2
cg02274033	3	0.0059288	0.109264757	5'UTR	opensea	TMEM108
cg14111928	10	0.0264851	0.108959155	5'UTR	opensea	MYST4
cg11471350	14	0.0203705	0.108955609	5'UTR	opensea	FOXN3
cg25932599	4	0.0005797	0.107738384	TSS1500	island	FGFRL1
cg23080845	6	0.0052873	0.102206343	5'UTR	opensea	ATXN1
cg02883147	12	0.0416931	-0.10321024	TSS200	shore	LOC144571
cg17052964	2	2.74E-05	-0.10651083	TSS1500	shore	SLC5A7
cg17977304	6	0.0058279	-0.10657196	TSS1500	opensea	MOG
cg00235754	15	3.27E-06	-0.10719532	5'UTR	opensea	CYFIP1
cg13244211	10	4.34E-06	-0.1093378	TSS1500	opensea	SNORA19
cg03006172	8	5.35E-09	-0.11013475	5'UTR	opensea	DLGAP2
cg18038361	18	7.53E-06	-0.11179072	TSS1500	opensea	TTR
cg26114124	12	0.0386413	-0.11193727	TSS200	island	LOC144571
cg10636144	8	0.0006324	-0.11951659	5'UTR	opensea	DLGAP2
cg15205441	10	1.38E-08	-0.12012278	5'UTR	opensea	CTBP2
cg25472172	2	2.90E-10	-0.17380845	5'UTR	opensea	GREB1
cg24509398	1	1.03E-11	-0.2733027	TSS1500	shore	EYA3

CPP and pre-pubertal controls

CG_ID	CHR	adj.P.Val	deltaBeta	feature	cgi	gene
cg17821500	1	0.005394	0.102336783	TSS1500	shore	AK2
cg24509398	1	0.0003493	-0.16228965	TSS1500	shore	EYA3

Figure 2: Correlation in beta-values between the CpGs in common with Bessa and the present study. The correlation has been performed between each group for each dataset.

CTPP vs CPP

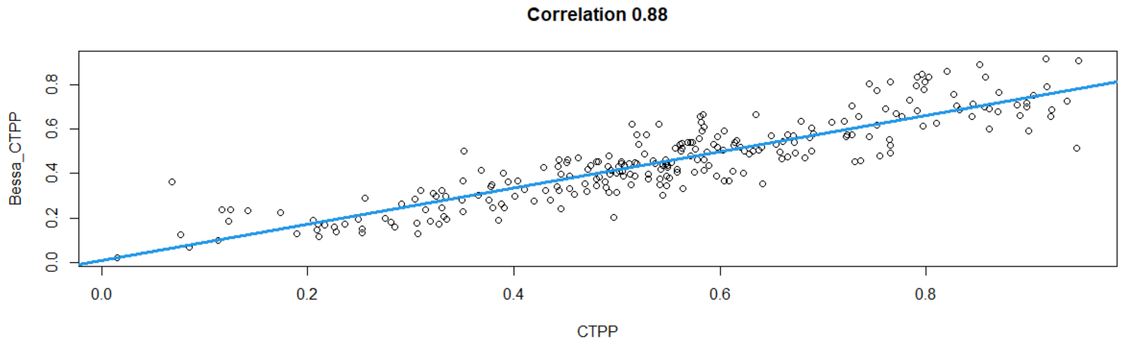

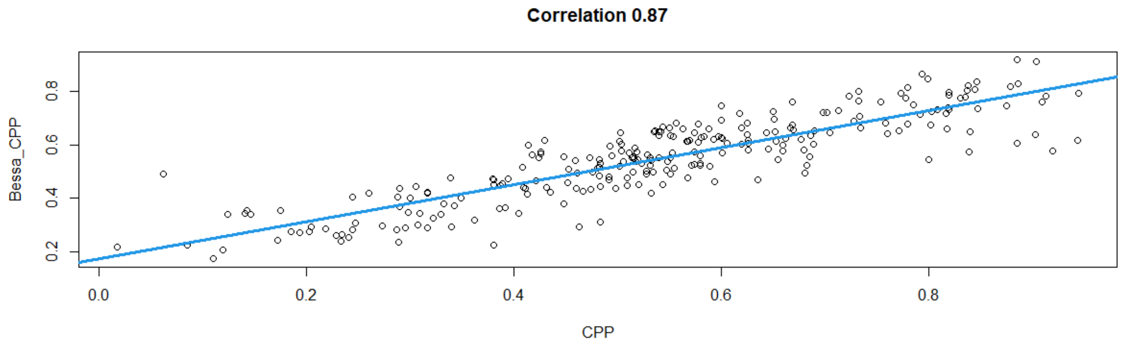

CTP vs CPP

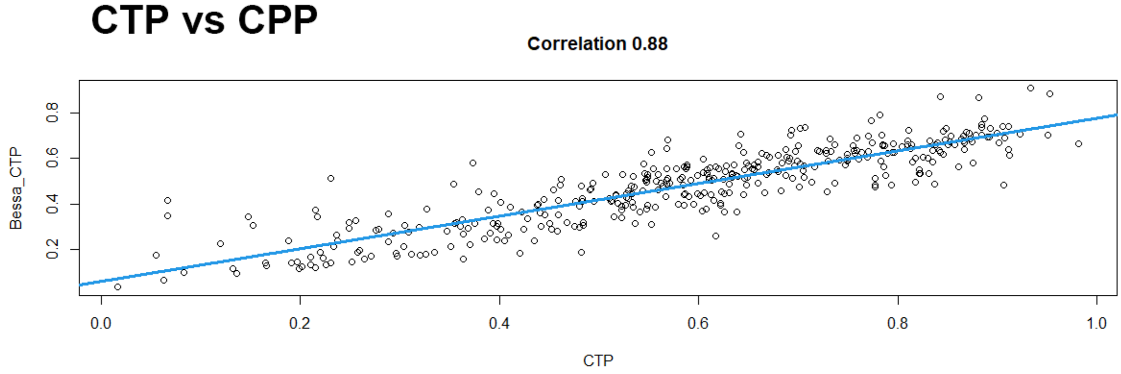

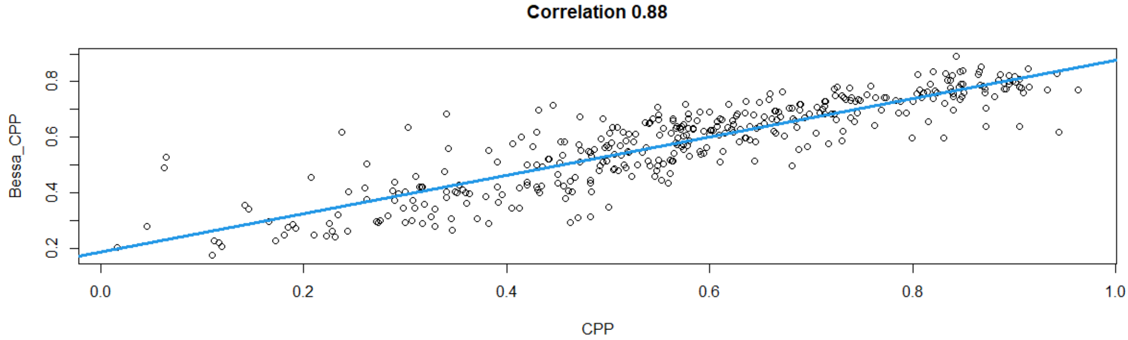

Supplement: Supplementary file 5 — Additional file 5: comparison with Bessa and Perry studies. [file 13148_2024_1683_MOESM5_ESM.pdf]
